# P-2058. Healthcare Utilization Before and After Enrollment in the New York City Health + Hospitals/Bellevue Health Equity Action Team, 2022-2024

**DOI:** 10.1093/ofid/ofaf695.2222

**Published:** 2026-01-11

**Authors:** Cooper Urban, Emma R Boockvar, Nicola Medrano, Jason Felder, Ashley Augustin, Dorothy Knutsen, Ofole Mgbako

**Affiliations:** New York University Grossman School of Medicine, North Java, NY; New York University Grossman School of Medicine, North Java, NY; New York University Grossman School of Medicine, North Java, NY; NYU Grossman School of Medicine, New York, NY; New York University Grossman School of Medicine, North Java, NY; NYU Langone, New York, New York; NYC Health+Hospitals, Brooklyn, NY

## Abstract

**Background:**

Patients hospitalized with HIV-related complications and substance use-related infections (e.g., endocarditis) often face sociostructural inequities that lead to poor health outcomes. Hospitalization is a key opportunity to address health-related social needs (HRSN) and reduce avoidable emergency department (ED) visits and readmissions. The Health Equity Action Team (HEAT), launched in July 2023 at NYC Health + Hospitals/Bellevue, provides inpatient consult and post-discharge navigation for patients with complex HRSN. HEAT leverages HRSN screening, trauma-informed care, peer support, and community partnerships to promote primary care utilization over ED or hospital-based care. We explored HEAT’s preliminary healthcare utilization outcomes before and after HEAT intervention.

**Figure:**
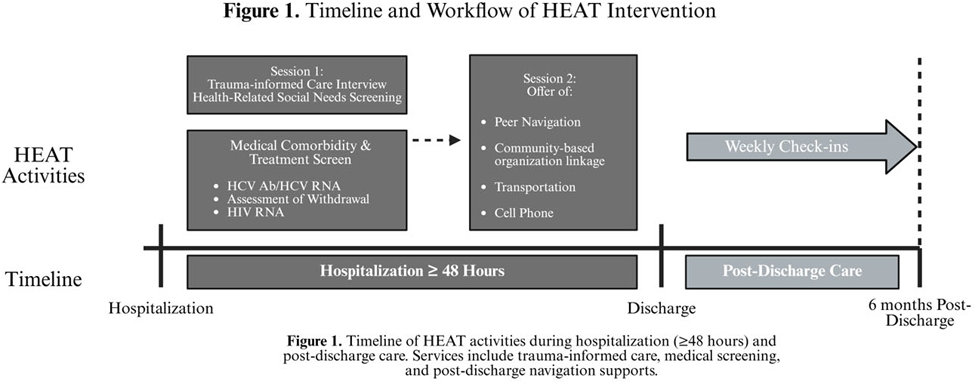

**Figure:**
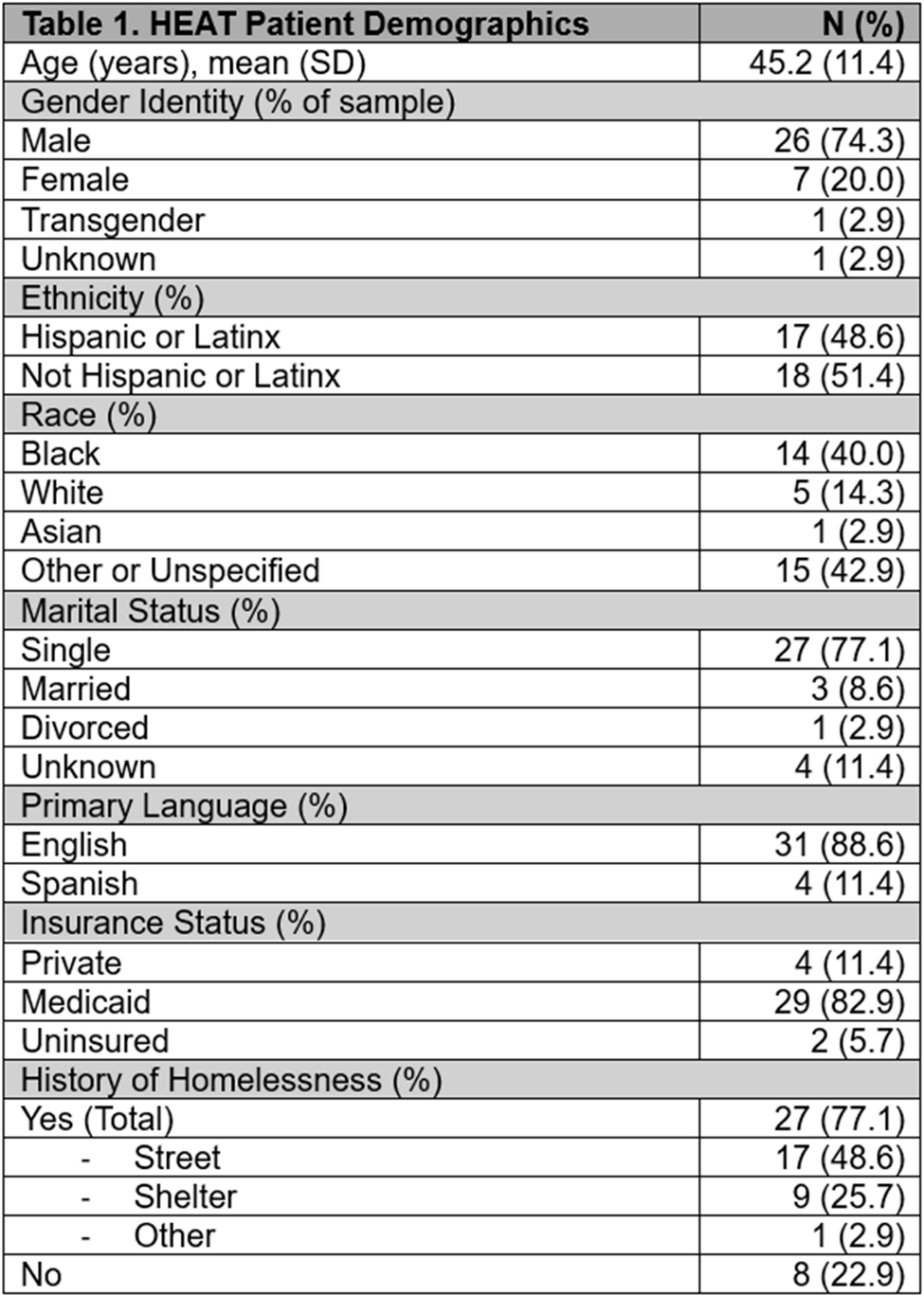

**Methods:**

We conducted a retrospective pre-post analysis of 35 patients who received the HEAT intervention (Figure 1) between January 1, 2022 and December 31, 2024. Manual chart review captured patient demographics, co-morbidities, and healthcare utilization six months pre- and post-HEAT consult. Outcomes included outpatient visits, ED visits, hospital admissions, and readmissions. We calculated means, standard deviations (SDs), and compared pre- and post-HEAT outcomes using paired t-tests at p < 0.05 as significant.

**Figure:**
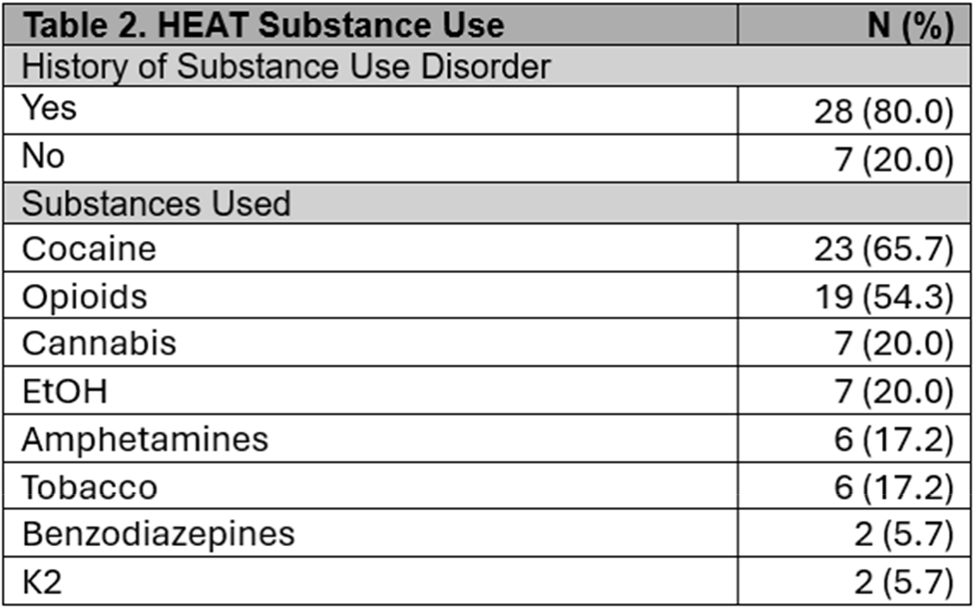

**Figure:**
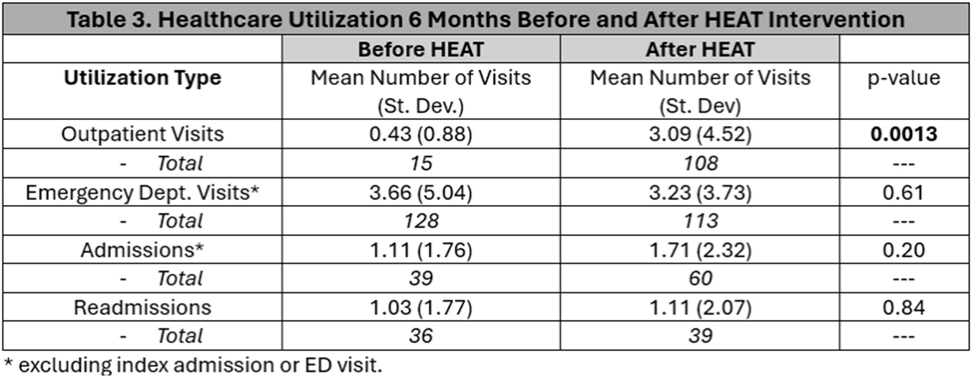

**Results:**

Participants were majority male (74%), Black (40%) or Latinx (49%), on Medicaid (83%), and experiencing homelessness (77%) (Table 1). Most (80%) had substance use disorder (SUD), 54% had HIV, 34% had both SUD and HIV, and 49% had hepatitis C. Substance use included primarily cocaine and opioids (Table 2). Mean outpatient visits from pre- to post-HEAT increased significantly (0.4 [SD 0.9] to 3.1 [SD 4.5], p < 0.01). ED visits remained stable (3.7 [SD 5.0] vs. 3.2 [SD 3.7], p = 0.61), as did hospital admissions (1.1 [SD 1.8] vs. 1.7 [SD 2.3], p = 0.20) and readmissions (1.0 [SD 1.8] vs. 1.1 [SD 2.1], p = 0.84).

**Conclusion:**

HEAT intervention was associated with increased outpatient follow-up among patients with HIV and SUD with historically low engagement in post-discharge primary care. Hospital and ED use remained stable. Future studies will assess HEAT’s long-term impact on healthcare utilization, quality, and safety.

**Disclosures:**

Ofole Mgbako, MD, MS, Gilead Sciences: Advisor/Consultant

